# Navigation-Assisted Total Knee Arthroplasty for Osteoarthritis with Extra-Articular Femoral Deformity and/or Retained Hardware

**DOI:** 10.1155/2013/174384

**Published:** 2013-09-24

**Authors:** Daisuke Hamada, Hiroshi Egawa, Tomohiro Goto, Tomoya Takasago, Michihiro Takai, Tetsuya Hirano, Yoshiteru Kawasaki, Natsuo Yasui

**Affiliations:** Department of Orthopedics, Institute of Health Biosciences, University of Tokushima, Graduate School, 3-18-15 Kuramoto, Tokushima 770-8503, Japan

## Abstract

Total knee arthroplasty (TKA) for osteoarthritis (OA) patients with extra-articular deformity is still challenging because angular deformity, canal sclerosis, or the retained hardware that precludes the use of the traditional intramedullary guide. In addition, atypical bone cut for intra-articular correction leads to imbalanced soft tissue gap. Furthermore, corrective osteotomy should be considered for severe deformity or para-articular deformity cases. Recently, navigation-assisted TKA has been reported to increase the accuracy of prosthetic positioning and limb alignment. This system can calculate mechanical axis regardless of extra-articular deformity, canal sclerosis, or retained hardware. Accordingly, navigation surgery has been considered to be a powerful option especially in TKAs with extra-articular deformity cases. Here, we report 3 successful navigation-assisted TKAs for osteoarthritis with extra-articular deformities and/or retained hardware. Navigation-assisted TKA is an effective and reliable alternative for patients with extra-articular deformities.

## 1. Introduction

The long-term success of total knee arthroplasty (TKA) is dependent on the accurate positioning of the prosthesis and proper soft tissue balancing. In most TKAs for arthritic knees, proper alignment and ligament balancing can be achieved by the techniques of appropriate bone resection and soft tissue release. On the other hand, TKA for knee arthritis with extra-articular deformity is still challenging based on the following reasons. (1) Altered mechanical axis due to angular deformity, canal sclerosis, or retained hardware which cannot be removed prevents the use of traditional intramedullary guide which is a useful tool for conventional TKAs. (2) Atypical intra-articular bone resection to perform intra-articular correction makes the soft tissue balancing complex. (3) Corrective osteotomy should be considered for severe deformity or para-articular deformity cases. 

Recently, computer-assisted navigation system has been used for TKA. A couple of studies have demonstrated that computer-assisted TKA provides more accurate, reliable, and reproducible component positioning [1, 2]. This procedure can make accurate bone resection regardless of angular deformity, canal stenosis, or retained hardware. Due to this, navigation surgery has been considered to be an effective option for extra-articular deformity cases.

In this paper, we report 3 successful navigation-assisted TKAs for osteoarthritis with extra-articular femoral deformities and/or retained hardware. 

## 2. Methods

 Long-standing radiographs were obtained from all patients preoperatively. After evaluating mechanical axis and angular deformity, we confirmed that the planned cutting line of the distal femur does not compromise the collateral attachment.

A single surgeon (DH) performed 3 consecutive TKAs in 2 patients (1 bilateral) with extra-articular femoral deformities and/or retained hardware using an image-free computer-based extramedullary guidance navigation system (Stryker Image Enhanced Knee Navigation Ver.2.0, Stryker, Kalamazoo, MI, USA). 

Under general anesthesia, a 15 cm anterior longitudinal skin incision was made, and standard medial parapatellar approach was used to expose the knee joint. After excision of the marginal osteophytes and cruciate ligaments, two pins were inserted in the distal femur and proximal tibia to connect infrared intraoperative device, which can send anatomical data to the computer continuously. Hip center registration was done first, followed by distal femoral registration. Positional information of the knee center, A-P axis, two epicondyles, and articular surfaces were used to determine the rotational alignment and mechanical axis. Next, we determined the tibia center, A-P axis, and medial and lateral plateau for proximal tibial registration. Ankle center was calibrated from medial and lateral malleoli. Mechanical axis was established using these data points.

Distal femoral cut was performed with the surface mapping guides of the image-free navigation system. Tracking devices were affixed to a universal positioning block. Bone resection was performed perpendicular to the mechanical axis in the coronal plane. Then, a posterior stabilized Scorpio NRG knee (Stryker Orthopedics) was tried. We evaluated the implant orientation, restored mechanical axis, and soft tissue balancing by the navigation system and clinically by the surgeon. Once a satisfactory mechanical axis and soft tissue balancing were obtained, all components were implanted with Amikacin-impregnated cement.

 Postoperative long-standing radiographs were also obtained 4 weeks after surgery to evaluate the restored mechanical axis. Clinical postoperative outcomes were assessed using Knee Society knee and function scores. The average follow-up period was 27.3 months (range, 17–40 months). 

## 3. Case report

### 3.1. Case 1: Malunited Femoral Shaft Fracture

**Figure 1:**
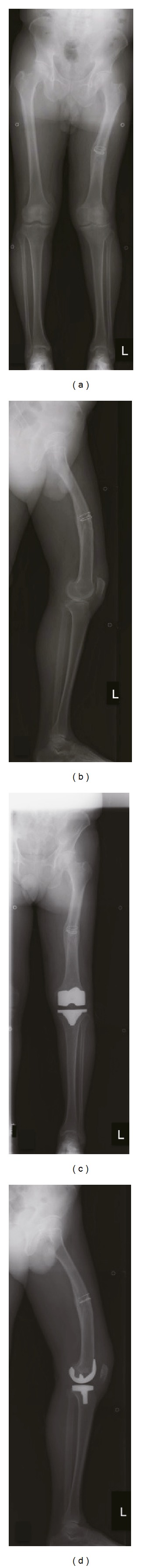
Case 1: preoperative anteroposterior standing radiograph (a) showed an extra-articular valgus deformity (11°) in the femoral shaft. Lateral view (b) revealed 23° antecurvatum deformity in the sagittal plane. Postoperative anteroposterior standing radiograph (c) demonstrated restored mechanical axis. In lateral view (d), femoral component was placed according to the intraoperative navigation data.

A 61-year-old man was involved in a motor vehicle accident at the age of 31. He sustained a left open femoral shaft fracture, and was treated with open reduction and internal fixation. Despite the treatment, the fracture was malunited and valgus deformity of the femoral shaft remained. Progressive left knee pain appeared 3 years before presentation. He was referred to us for surgery. Radiographs showed advanced degenerative arthritis of the left knee and malunited left femoral shaft fracture (Figures 1(a) and 1(b)). There was a 11° valgus in coronal plane and 23° antecurvatum deformity in sagittal plane preoperatively.

Extra-articular deformity and canal sclerosis precluded the use of a conventional intramedullary alignment guide; therefore, we performed navigation-assisted TKA. 

**Figure 2:**
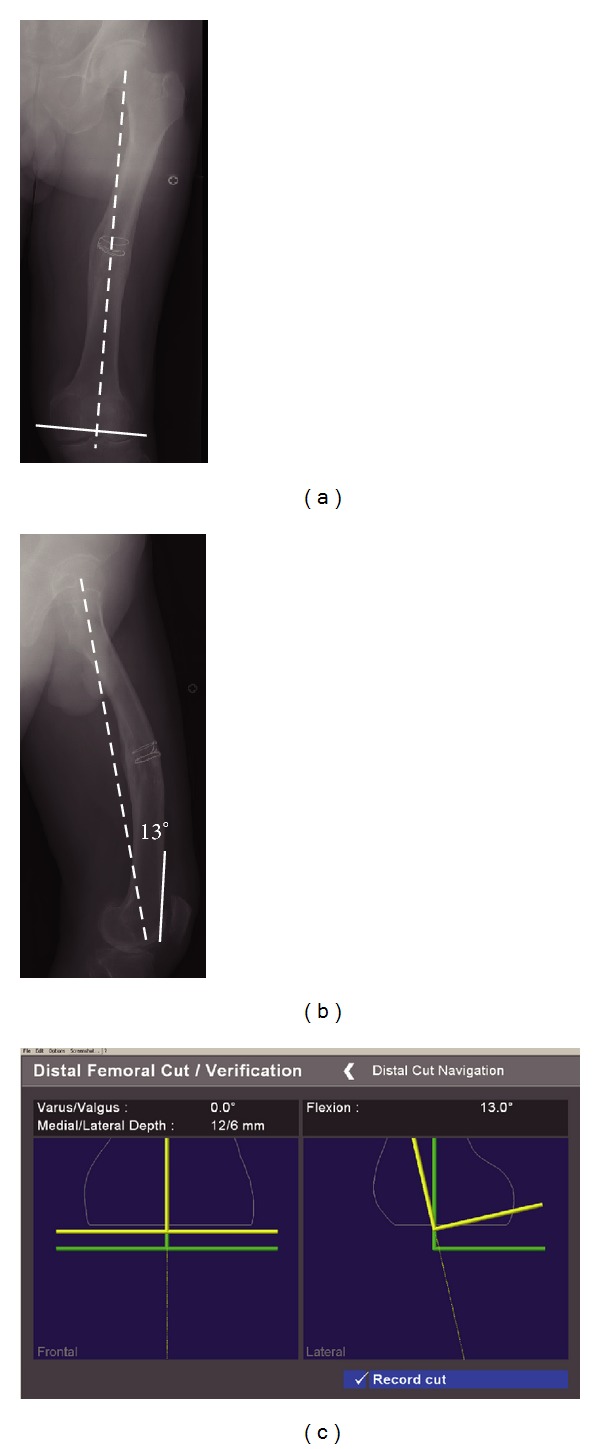
Estimated distal femoral cut line (solid line) perpendicular to the mechanical axis (dashed line) does not compromise the collateral attachment in anteroposterior radiograph (a). Anterior femoral surface (solid line) is 13° flextion to the mechanical axis (dashed line) in lateral view (b). Intraoperative estimation of the distal femoral cut line and thickness of the resected bones were calculated by the navigation system (c).

Preoperative planning revealed that the estimated distal femoral cut line does not compromise MCL insertion site (Figure 2(a)). The distal femoral cut line was set 13° flexion to the mechanical axis in the sagittal plane (Figure 2(b)) to avoid notching of the anterior femoral cortex. According to the intraoperative assessment, the estimated thickness of distal femoral resection was 12 mm for the medial condyle and 6 mm for the lateral condyle (Figure 2(c)). 

Postoperative radiographs showed well-restored coronal limb alignment. Coronal alignment of the femoral component was 90° to the mechanical axis (Figure 1(c)). Sagittal alignment was 13° flexion to the mechanical axis that is parallel to the anatomical axis of the distal femur (Figure 1(d)). The femoral component was placed according to the intraoperative navigation data. Mechanical axis of the lower limb improved from 9° valgus preoperatively to 1° varus postoperatively. 

Range of motion improved from 15°–105° to 10°–120°. Knee score increased from 36 to 92, and function score increased from 50 to 90 at the latest follow-up. Soft tissue balance was well coordinated. No complications, such as infection, deep venous thrombosis, PF problems, or aseptic loosening, were seen postoperatively. As a result, the leg length discrepancy became smaller after restoring the limb alignment.

### 3.2. Case 2: Malunited Supracondylar Fracture Retaining Hardware

**Figure 3:**
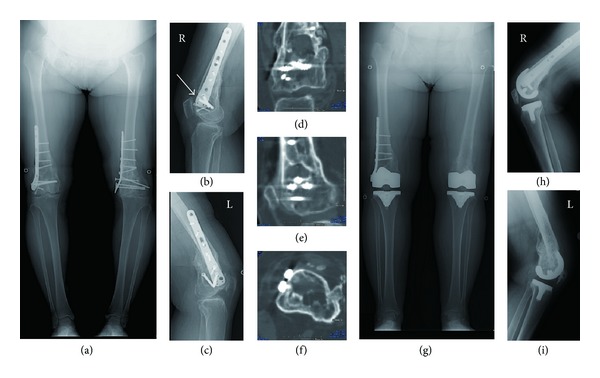
Case 2: preoperative anteroposterior standing radiograph (a) showed both malunited supracondylar fracture with hardware. Arrow on the lateral view of the right knee (b) indicates the defect of anterior cortex. Lateral view of the left knee (c) showed displaced distal femur. Coronal (d), sagittal (e), and axial (f) CT images of the right knee revealed the large cavity at the fracture site. Postoperative anteroposterior standing radiograph (g) demonstrated restored mechanical axis. Lateral view of the right knee (h) and the left knee (i) showed accurately placed prosthesis.

A 59-year-old woman presented with both knee pain and contracture of the left knee, which resulted in functional disability. She was not able to walk without 2 crutches preoperatively. Four years before presentation, she fell from a second-floor balcony due to epileptic attack and sustained open fracture of both knees. She was transported to the emergency room and underwent open reduction and internal fixation of both femurs. These fractures were healed without infection; however, there remained malunions in both femurs. Progressive pain in both knees leading to limping and stiffness developed gradually. She was referred to our hospital for surgery. Radiographs showed advanced degenerative arthritis of both knees, extra-articular deformity after supracondylar fracture and retained hardware (Figure 3(a)). The magnitude of angular deformity of the right femur was rather small (Figure 3(b)); however, there was a large cavity filled with fibrous tissue and partial defect of the anterior cortex in the distal femur (Figures 3(b), 3(d), 3(e), and 3(f)). On the other hand, the distal segment of the left femur was displaced laterally and posteriorly, and the overlap at the fracture site resulted in leg length discrepancy (Figure 3(c)).

 Firstly, we performed right TKA because her right knee pain was more severe compared to her left knee. To avoid refracture due to partial defect of the anterior femoral cortex, we decided to place the femoral component without removing the hardware using navigation system. Preoperative planning confirmed that hardware was not likely to interfere with the femoral component.

After removal of the Herbert screws, distal femoral cut was performed perpendicular to the mechanical axis in the coronal plane and 3° flexion to the mechanical axis in the sagittal plane using navigation system. Cancerous bone chips made from resected bones were grafted into the cavity after clearing up the fibrous tissue.

Six months later, she underwent left TKA. Preoperative radiographs showed not only bone union but also loose screws, so we determined to remove the hardware. Laterally and posteriorly shifted distal femur prevented the use of the traditional intramedullary guide. Therefore, we used navigation system again. Distal femur was resected at a right angle to the mechanical axis in coronal plane and 3° flexion to the mechanical axis in the sagittal plane.

Postoperative radiographs demonstrated good positioning of the components and restored mechanical axis (Figures 3(g), 3(h), and 3(i)). Mechanical axis of the lower limb improved from 9° varus to 2° varus for the right knee and from 13° varus to 0° for the left knee.

Range of motion improved from 5°–90° to 0°–95° for right knee and from 10°–30° to 0°–75° for left knee. Knee score improved from 31 to 84 for the right knee and from 25 to 80 for the left knee. The function score improved from 20 to 70 at the latest follow-up. Soft tissue balance was well coordinated. No complication was seen postoperatively.

## 4. Discussion

In TKA for extra-articular deformity cases, surgeons have two options: one is intra-articular correction using TKA and the other is TKA with simultaneous or staged corrective osteotomy. So firstly, we should consider whether the altered mechanical axis can be restored by intra-articular bone resection or not.

Basically, intra-articular correction is indicated if the estimated distal femoral cut line does not compromise the collateral attachment [3]. In coronal plane deformity, the impact of the deformity becomes greater as its apex approaches the knee joint [4]. Accordingly, the feasibility of intra-articular correction is determined by the degree of the deformity and the distance of the deformity from the knee. Only J.-W. Wang and C.-J. Wang noted the limitation of the deformity in their successful series of conventional TKA for extra-articular varus deformity of 20° or less [5]; however, there is no critical limitation to the degree of extra-articular deformity in coronal plane that can be restored by intra-articular correction. Thus, careful preoperative evaluation is still important for decision [6]. If intra-articular correction is not feasible, simultaneous or staged corrective osteotomy should be considered.

In the first case presented, there was a 11° valgus deformity in the femoral shaft that was not close to the knee joint, although canal sclerosis appeared at 11 cm proximal from the condylar surface. In the left knee of the second case, the distal femur shifted laterally and posteriorly. Thus traditional intramedullary guide cannot be used in such cases [7]. Although a short intramedullary guide or extramedullary guide might be available, they might lead to a less predictable clinical and radiographic outcomes. On the other hand, image-free computer-assisted navigation system can calculate the mechanical axis using the femoral head center, the center of the knee joint, and the center of the ankle regardless of the angular deformity and/or canal stenosis [6]. In addition, continuous feedback from the navigation system allows for intraoperative evaluation of the mechanical axis [8] and correction of the cutting error during surgery [9]. Furthermore, this procedure reduces the risk of excessive bone cuts, blood loss, and fat embolism. Klein et al. first reported intra-articular correction of extra-articular deformity by navigation-assisted TKA [7]. Since then, several reports that achieved good function and alignment outcomes in osteoarthritis with extra-articular deformity have been published [6, 10–14].

In terms of extra-articular femoral deformity in sagittal plane, Wang et al. reported intra-articular correction with TKA for femoral deformity of up to 15° recurvatum and up to 16° antecurvatum in sagittal plane [15]. In our first case, the sagittal angulation was 23° antecurvatum. Preoperative evaluation from the long-standing lateral radiograph revealed that the distal femoral cut perpendicular to the mechanical axis in sagittal plane would result in anterior notching. Therefore, distal femoral bone resection was performed 13° flexion to the mechanical axis, which is perpendicular to the distal femoral axis, for anatomical installation of the prosthesis to the displaced distal femur in sagittal plane. In this procedure, surgeons can control bone resection to avoid anterior femoral notching. This variance in performance is also an excellent benefit of the navigation system.

Because hip motion can compensate the modest deformity in the sagittal plane, it might not have major impact compared to the deformity in the coronal plane. Therefore, bone resection perpendicular to the sagittal mechanical axis is not necessary. The femoral component should be installed in accordance with the anatomical axis of the distal femur, rather than mechanical axis in sagittal plane.

The authors were not aware of any articles that note limitations of the rotational deformities. The limitation of intra-articular correction is that the leg-length discrepancy or rotational deformity cannot be corrected by this procedure. If a patient has a considerable leg-length discrepancy or unacceptable rotational deformity, limb lengthening or corrective osteotomy should be considered.

Navigation-assisted TKA is also an effective tool for the cases with hardware [6, 7, 10]. In the right knee of our second patient, the distal end of the plate was close to the joint. We could place femoral component without interference. If prosthesis has the possibility to interfere with the retained plate that cannot be removed, CT-based 3D templating might be an additional option.

Although long-term follow-up is not available, postoperative course is uneventful. Further, follow-up is needed.

In summary, we demonstrated navigation-assisted TKAs with angular deformity and/or hardware of the femur successfully and safely. In cases of extra-articular deformity, careful preoperative assessment should be necessary. If intra-articular correction is applied, the navigation system allows us to make precise bone resection and fine restored mechanical alignment. The advantage of the navigation-assisted TKA for normal cases remains a matter of debate; however, navigation-assisted TKA is an excellent alternative for extra-articular deformity and/or hardware retaining cases.

## References

[1] Mullaji A, Kanna R, Marawar S, Kohli A, Sharma A (2007). Comparison of limb and component alignment using computer-assisted navigation versus image intensifier-guided conventional total knee arthroplasty: a prospective, randomized, single-surgeon study of 467 knees. *Journal of Arthroplasty*.

[2] Kim SJ, MacDonald M, Hernandez J, Wixson RL (2005). Computer assisted navigation in total knee arthroplasty: improved coronal alignment. *Journal of Arthroplasty*.

[3] Lonner JH, Siliski JM, Lotke PA (2000). Simultaneous femoral osteotomy and total knee arthroplasty for treatment of osteoarthritis associated with severe extra-articular deformity. *Journal of Bone and Joint Surgery A*.

[4] Wolff AM, Hungerford DS, Pepe CL (1991). The effect of extraarticular varus and valgus deformity on total knee arthroplasty. *Clinical Orthopaedics and Related Research*.

[5] Wang J-W, Wang C-J (2002). Total knee arthroplasty for arthritis of the knee with extra-articular deformity. *Journal of Bone and Joint Surgery A*.

[6] Mullaji A, Shetty GM (2009). Computer-assisted total knee arthroplasty for arthritis with extra-articular deformity. *Journal of Arthroplasty*.

[7] Klein GR, Austin MS, Smith EB, Hozack WJ (2006). Total knee arthroplasty using computer-assisted navigation in patients with deformities of the femur and tibia. *Journal of Arthroplasty*.

[8] Shao J, Zhang W, Jiang Y (2012). Computer-navigated TKA for the treatment of osteoarthritis associated with extra-articular femoral deformity. *Orthopedics*.

[9] Manzotti A, Cerveri P, de Momi E, Pullen C, Confalonieri N (2010). Relationship between cutting errors and learning curve in computer-assisted total knee replacement. *International Orthopaedics*.

[10] Fehring TK, Mason JB, Moskal J, Pollock DC, Mann J, Williams VJ (2006). When computer-assisted knee replacement is the best alternative. *Clinical Orthopaedics and Related Research*.

[11] Bottros J, Klika AK, Lee HH, Polousky J, Barsoum WK (2008). The use of navigation in total knee arthroplasty for patients with extra-articular deformity. *Journal of Arthroplasty*.

[12] Chou WY, Ko JY, Wang CJ, Wang FS, Wu RW, Wong T (2008). Navigation-assisted total knee arthroplasty for a knee with malunion of the distal femur. *Journal of Arthroplasty*.

[13] Kim KI, Ramteke AA, Bae DK (2010). Navigation-assisted minimal invasive total knee arthroplasty in patients with extra-articular femoral deformity. *Journal of Arthroplasty*.

[14] Tigani D, Masetti G, Sabbioni G, Ben Ayad R, Filanti M, Fosco M (2012). Computer-assisted surgery as indication of choice: total knee arthroplasty in case of retained hardware or extra-articular deformity. *International Orthopaedics*.

[15] Wang J-W, Chen W-S, Lin P-C, Hsu C-S, Wang C-J (2010). Total knee replacement with intra-articular resection of bone after malunion of a femoral fracture: can sagittal angulation be corrected?. *Journal of Bone and Joint Surgery B*.

